# What Serious Video Games Can Offer Child Obesity Prevention

**DOI:** 10.2196/games.3480

**Published:** 2014-07-16

**Authors:** Debbe Thompson

**Affiliations:** ^1^USDA/ARS Children's Nutrition Research CenterPediatricsBaylor College of MedicineHouston, TXUnited States

**Keywords:** serious video games, children, teenagers, obesity prevention, formative research, qualitative research

## Abstract

Childhood obesity is a worldwide issue, and effective methods encouraging children to adopt healthy diet and physical activity behaviors are needed. This viewpoint addresses the promise of serious video games, and why they may offer one method for helping children eat healthier and become more physically active. Lessons learned are provided, as well as examples gleaned from personal experiences.

## Childhood Obesity

Childhood obesity is a worldwide public health issue [1,2], and is associated with adverse health effects in both children and adults [3,4]. Obese children and adolescents are more likely to become obese adults, further exacerbating the public health urgency of this issue [3,5]. Diet and physical activity behaviors, the cornerstones of obesity prevention [6,7], track into adulthood [8,9]. Establishing healthy diet and physical activity behaviors during childhood is an important aspect of obesity prevention. Effective ways to achieve and maintain this goal are needed.

Technology may offer an effective method for encouraging children to adopt healthy behaviors. Technology is pervasive in today’s world and influences many aspects of our lives, including how we communicate, educate, and entertain ourselves. Video games are an example; 97% of 12-17 year olds play video games, play multiple genres, and have access to computers and other devices on which to play the games [10,11]. Internet access, including high-speed access [11], has increased, making Internet games more accessible. Evidence indicates the digital divide is diminishing [12], particularly when mobile platforms are taken into account [13,14]. Thus, Internet video games offer a familiar, convenient, and readily accessible channel within which to evaluate obesity prevention programs developed for children and teenagers.

While traditional video games are primarily designed to entertain, a new genre has emerged that seeks to do more. Serious video games, often referred to as games for health when they target health behaviors [15], are showing promise as a method for promoting healthy behaviors to youth [15,16]. Serious video games have the dual goal of entertaining, while promoting behavior change [17,18]. This game genre has the formidable task of achieving a balance between “fun-ness” (ie, components that entertain, such as animation, storyline, sound effects) and “serious-ness” (ie, the components that promote behavior change, such as goal setting, problem solving) [18]. This balance, although sometimes difficult to attain, is essential; too much “fun-ness”, the game will entertain, but it will not likely change behavior; on the other hand, too much “serious-ness”, and the players will not likely play all levels or episodes of the game (ie, inadequate dose), making behavior change less likely.

## Serious Game Components Targeting Behavior Change

There are several components that should be considered when developing serious games for health behavior change. These include interactivity, feedback, characters, story, and sound effects [18,19]. For examples of how features such as these were incorporated into a serious video game, please see Thompson et al [18].

### Interactivity

Rather than a didactic presentation of facts or figures, the player is able to interact with and “discover” knowledge and skills, and then immediately apply these to overcoming behavioral obstacles in the game.

### Feedback

A serious video game offers a unique opportunity to receive immediate feedback, thus refining, reinforcing, and enhancing newly found knowledge and skills.

### Characters

The protagonists model desired behaviors, while the antagonists attempt to “foil” the player’s efforts to attain a goal, thus adding challenge and interest.

### Story

The story adds intrigue and excitement, and can be designed to “wrap” the behavioral components into the game for a more seamless experience.

### Sound Effects

The sound effects convey meaning and direction without words. For example, a ticking clock or fast-paced music can indicate that time is running out or that the player must act quickly.

My colleagues and I have been involved in the development and evaluation of a variety of serious video games and other technology-based interventions for a number of years [20-33]. We have learned many lessons along the way, including the following.

## Just Because You Can, Does Not Mean You Should

Think about the behavior you’re targeting and the project goal. It may be that another method or approach would be a better fit.

For example, research suggests that video games may not be the best method for enhancing moderate to vigorous physical activity [34,35]. We have seen this in our own work [20,21]. It is highly likely that we have not found the “ideal” method for using technology to promote physical activity. This is an area that needs additional investigation. For example, we are investigating the effect of text messages [33] and avatars created from photographs of the player (ie, self-representational avatars) as ways to promote physical activity to teenagers. It may be that traditional approaches are more effective than serious video games at enhancing physical activity; however, before we give up on technology as a way to promote physical activity, it is imperative that additional methods, such as these and others, be investigated.

## Know What You Want Before You Start

Once programming has been completed, it is expensive to make a change. An approach that has worked for us is to know what you want and how the pieces fit together before starting development. For example, first identify the “serious” components that need to be included based on the theoretical framework (ie, content, behavioral procedures) and the targeted behavior (ie, diet, physical activity) [19]. Then negotiate with the design studio about how to structure the game to create a seamless, entertaining experience for the player. We have discovered that following a process similar to this minimizes miscommunication and increases the likelihood that you will be satisfied with the end result [19].

## Carefully Assess Your Budget

Serious video games can be expensive to develop [19]. In addition to having an ample budget to cover the costs of development, it is wise to have a contingency budget to cover unexpected issues (eg, technical problems, etc) that may develop. Often, it is not “if” unexpected delays or set backs will occur, but “What do I do now?” when they do. Anticipate and plan for them.

We have seen this in our own technology-based work. Negotiation and open communication with the game design team are essential. Using the approach described above has been extremely beneficial to achieving our research goals within a reasonable budget. For example, in a serious video game promoting fruit and vegetables to youth [31], during discussions with the game design studio, we were informed that flickering torches were possible, but it would be expensive to animate them, which was not feasible given our budget. Through negotiation and communication, we compromised on a lower cost way to animate the torches (ie, overlays versus actual animation), leaving us both satisfied with the outcome. Other issues that need to be discussed with the game design studio are commercialization and intellectual property rights (ie, who owns what) [19].

## Partner With Your Target Audience

The importance of research with the target audience to serious video game design cannot be emphasized enough [36]. It is important to understand their perspective before initiating game development. For example: (1) What are their beliefs and attitudes regarding this behavior?; (2) What problems do they face when trying to engage in the behavior?; (3) What type of information do they want, and in what format?; (4) What would a serious video game addressing this behavior need to include?; and (5) equally as important, What should it not include? [32,36]. Alpha testing with them throughout development is also important, for example: (1) Did we get it right?; (2) What changes, if any, are needed?; (3) Do you understand what this is asking you to do?; and (4) Can you do it using the approach presented in the serious video game?. Beta testing to identify technical issues (ie, “bugs” or “glitches”) before finalizing the game is also essential. During this phase, be sure to test the serious video game with different operating systems, hardware, browsers, and connection speeds. After the outcome evaluation has been completed, asking the target audience, (“Did we meet your expectations?” and “What, if anything, should we do differently next time?”), would inform the next game you develop. Partnering with the target audience is essential. Partnering with them will help avoid costly mistakes and help ensure the game addresses their needs and interests in an appealing and acceptable manner.

We used this approach when developing a serious video game for elementary aged school children [31]. We first convened an expert panel of 4th and 5th graders, and, over a period of several months, they vetted critical aspects of the serious video game, such as characters, storyline, and behavior change components. Several of the behavioral components involved complicated concepts, such as implementation intentions (ie, plans connected to goal attainment) [37]. Therefore, it was essential to obtain the children’s feedback on whether they understood what they were being asked to do and whether they could do what they were being asked to do. Their comments were used to refine the game components as identified above and enhance its developmental appropriateness and appeal. They also participated in a pilot test, which served as a final beta test of the serious video game and its procedures.

## Do Not Forget About the Data

A myriad of data can be collected as players navigate through the game, but not all of it may be useful (eg, time spent in the serious video game, which may not be useful if gameplay is discontinued for another activity, such as eating dinner, but the game or Web browser is not closed). Identify in advance what data you want to capture (eg, goals set, goals attained, problems encountered, time needed to attain each level) early in the development process. Talk with the game design studio about the data you need and how you would like these data stored in the back-end database to avoid potentially expensive (or unfortunate) surprises at the end (ie, miscommunications regarding which data were to be collected and/or how they were to be stored). Data security is also an important issue that should be addressed, (ie, How will the data, as well as confidentiality, be protected?).

When we develop technology-based programs, we typically have a discussion with the game design studio well before the end of the programming phase, where we discuss options for data collection, and how the data are stored in the database. We have learned that this process helps to avoid missteps and opens up an important discussion between key players. For example, when developing the serious video game promoting fruit and vegetable consumption [31], we provided the game design studio with a document that identified the data we needed and how the data tables should be formatted, including whether the tables needed to include numbers or words (eg, “choice 2” or “carrots”). We then had a discussion about possible options and came to an agreement about what data would be collected, and what the tables would look like. During this discussion, it was discovered that the game design studio and the research team had very different opinions about how the game play information would be saved. This realization proved to be critical and opened up an extremely important dialogue between the game design studio and the research team. This process made several key points very clear-do not make assumptions; keep the lines of communication open; and be open to alternative ways of accomplishing the same goal-you may be pleasantly surprised by the result.

Developing serious video games is a difficult, time consuming, humbling, and exhausting experience, but it is also exhilarating. When a parent tells you what a difference the game has made in his or her child’s life, or the outcome evaluation shows the child not only played all episodes of the game, but modified their behavior, such as eating more fruit and vegetables [21], you realize the potential of this work for child obesity prevention. Will you make mistakes? Yes; like most things in life, there is no iron clad formula for success. But when you get it right, it is invigorating, and you can’t wait to start working on the next serious video game. There is nothing like knowing that you made a difference in someone’s life, particularly a child’s life.

Relax, and enjoy the journey! In my opinion, it’s well worth it.

## Abbreviations

ARS: Agriculture Research Service
USDA: United States Department of Agriculture
